# Editorial

**Published:** 1871-02

**Authors:** 


### Editorial.

THE SOCIAL EVIL.—A large amount of space in the present number of the *Examiner* is occupied by the article of Prof. Andrews, presenting a most interesting and full consideration of this subject. During the last five years a strong effort has been made to get the subject before the Legislatures of the several States; and many are advocating the adoption of measures similar to those already in operation in many of the European cities, by which prostitution is legally recognized under the pretense of *regulation.*

Inasmuch as members of our profession must become active agents in the carrying out of any measures designed to lessen the evils of prostitution, and may exert great influence in shaping legislation on the subject, they should be fully and correctly informed concerning the results of such efforts as have already been made. For this purpose, the article of Prof. Andrews will be found of great value. Individually, we have no faith in any measures for lessening or eradicating the *Social Evil*, that are not applied just as rigorously to the *male* as the female.

MEDICAL COLLEGE COMMENCEMENTS.—The Commencement Exercises of Rush Medical College will take place on Wednesday, Feb. 1, 1871. The annual meeting of the Alumni Association of that College will be held in the lecture-room of the College, at 10 o'clock A.M. of the same day. Annual address by Abner Hard, M.D., of Aurora.

The Commencement Exercises of the Chicago Medical College, Medical Department of N. W. University, will be held in the College Hall on Tuesday afternoon, March 14, 1871.

The annual meeting of the Alumni Association of the College will be held in the same place, and on the same day, at 10 o'clock A.M. Annual address by the President of the Association.

Hydrate of Chloral—Large Dose—Recovery.—A short time since, in this city, a lady who was suffering severely with an attack of neuralgic pain in the head, in a moment of mental bewilderment, took a vial full of a solution of chloral at once, amounting to 240 grains. The medicine had been directed only an hour or two previous by her attending physician, and was to have been taken in doses of one teaspoonful at suitable intervals. The enormous dose was immediately followed by complete insensibility and great depression of respiration and circulation. The physician did not reach her until so much time had elapsed, that it was probable the whole amount had been absorbed; consequently, no vomiting was, or could be, induced. For eighteen hours she appeared every moment in danger of dying from suspended respiration, and during this time manifested no sign of consciousness. The continuance of respiration was aided by galvanic currents, kept up most of the time until the effects of the medicine began to abate.

After eighteen hours the respiration began to be more regular, and the patient more restless, and, at the end of twenty-three hours, she had improved in consciousness so far as to make efforts to speak, and from that time went on to complete recovery. This case shows that there must be either great difference in the quality of hydrate of chloral in our drug stores, or great variations in the capacity of different patients to sustain life under its effects.

---

Large Brain.—Dr. J. B. Woodson, of Kansas City, Mo., writes us that he recently removed from a human skull a brain in a perfectly healthy condition, that weighed 60 ounces, which is near the highest weight of human brain on record. But the Doctor does not inform us whether the owner of the brain, while living, was a statesman, philosopher, or fool.

---

RELIABLE VACCINE MATTER CAN BE HAD OF
DR. S. A. McWILLIAMS,
166 STATE STREET, CHICAGO.

### Mortality for the Month of December, 1870.

| | |
|---|---|
| Accidents, Asphyxia | 1 |
| " burned in building | 1 |
| " by fall | 2 |
| " railroad | 2 |
| " suffocation | 2 |
| " scalded | 3 |
| Abscess | 1 |
| Apoplexy | 10 |
| Asthma | 3 |
| Bowels, congestion of | 1 |
| " ulceration of | 1 |
| Brain, congestion | 3 |
| " compression of | 1 |
| " inflammation | 5 |
| " softening | 4 |
| " effusion in | 1 |
| Bronchitis | 9 |
| " capillary | 2 |
| " chronic | 2 |
| Cancer of liver | 3 |
| " stomach | 2 |
| " uterus | 1 |
| " breast | 1 |
| " bowels | 1 |
| " pancreas and bladder | 1 |
| Child birth | 1 |
| Chest, tumor of | 1 |
| Cholera Infantum | 1 |
| Consumption | 33 |
| Convulsions | 38 |
| " puerperal | 3 |
| Croup | 27 |
| Debility general | 3 |
| Delirium Tremens | 1 |
| Diarrhœa | 1 |
| " chronic | 1 |
| Diphtheria | 15 |
| Dropsy | 1 |
| " abdomen | 1 |
| " renal | 1 |
| Dysentery | 1 |
| " chronic | 1 |
| Embolia | 1 |
| Entero colitis | 1 |
| Enteritis | 6 |
| Epilepsy | 2 |
| Erysipelas | 2 |
| Fever, congestive | 1 |
| " puerperal, | 4 |
| " remittent | 3 |
| " scarlet | 7 |
| " & complications | 3 |
| " malignant | 1 |
| " typhoid | 18 |
| Gastritis | 5 |
| Gastro enteritis | 3 |
| Hemorahage, umbilical | 1 |
| Heart disease | 4 |
| " hypertrophy of | 1 |
| " inflammation | 2 |
| " organic disease of | 4 |
| " valvular disease | 4 |
| Hepatitis, | 1 |
| Hernia, strangulated | 1 |
| Hip-disease | 1 |
| Hydrocephalus | 5 |
| " acute | 3 |
| Inanition | 10 |
| Kidneys, Bright's disease | 1 |
| Laryngitis | 5 |
| Liver, rupture of | 1 |
| Lungs, congestion | 7 |
| " hemorrhage | 1 |
| Manslaughter | 2 |
| Measles | 1 |
| " and complications | 2 |
| Meningitis | 9 |
| Myetitis | 1 |
| Nervous shock, surgical operation | 1 |
| Old age | 15 |
| Paralysis | 3 |
| Peritonitis | 3 |
| Peritonaeum inflammtion of | 1 |
| Pneumonia | 34 |
| " typhoid | 4 |
| Pyaemia | 3 |
| Rheumatism | 1 |
| Scrofula | 3 |
| Suicide by chloroform | 1 |
| " poison | 1 |
| " shooting | 1 |
| Syphilis, secondary | 1 |
| Tabes mesenterica | 10 |
| Teething | 2 |
| " and complications | 3 |
| Tetanus | 2 |
| Uterus, hemorrhage of | 1 |
| " ulcer of | 1 |
| Whooping-cough | 4 |
| Total | 415 |

### AGES.

| | |
|---|---|
| Under 1 | 108 |
| 1 to 2 | 51 |
| 2 to 3 | 13 |
| 3 to 4 | 21 |
| 4 to 5 | 10 |
| 5 to 10 | 23 |
| 10 to 20 | 19 |
| 20 to 30 | 38 |
| 30 to 40 | 35 |
| 40 to 50 | 29 |
| 50 to 60 | 20 |
| 60 to 70 | 23 |
| 70 to 80 | 17 |
| 80 to 90 | 8 |
| 90 to 100 | |
| Total, | 415 |

| | | | | | |
|---|---|---|---|---|---|
| Males, | 240 | Females, | 175 | Total, | 415 |
| Single, | 278 | Married | 137 | Total, | 415 |
| White, | 412 | Colored, | 3 | Total, | 415 |

### COMPARISON.

Deaths in 1870, Dec., 415 | Deaths in 1869, Dec., 440 | Decrease, 25

Deaths in Nov., 1870, 427 | Decrease, 12

### NATIVITY.

| | | | | | |
|---|---|---|---|---|---|
| Austria | 1 | Denmark | 1 | Norway | 5 |
| Bohemia | 3 | England | 8 | Nova Scotia | 1 |
| British Columbia | 1 | France | 1 | Russia | |
| Canada | 7 | Germany | 55 | Switzerland | 2 |
| Chicago, Native | 65 | Holland | 2 | Scotland, | 10 |
| Chicago, Foreign | 127 | Ireland | 44 | Sweden | 12 |
| U. S., other parts | 65 | Italy | 1 | Unknown | 4 |
| | | | | Total, | 415 |

### MORTALITY BY WARDS FOR THE MONTH.

| Wards. | Mortality. | Wards. | Mortality |
|---|---|---|---|
| 1 | 7 | 18 | 27 |
| 2 | 18 | 19 | 13 |
| 3 | 16 | 20 | 20 |
| 4 | 7 | Accidents | 11 |
| 5 | 6 | Bridewell | 1 |
| 6 | 34 | County Hospital | 10 |
| 7 | 13 | Erring Woman,s Refuge | 1 |
| 8 | 22 | Home for Friendless | |
| 9 | 35 | Half Orphan Asylum | |
| 10 | 16 | Marine Hospital | 1 |
| 11 | 22 | Manslaughter | 2 |
| 12 | 16 | Mercy Hospital | 3 |
| 13 | 9 | Protestant Orphan Asylum | |
| 14 | 10 | St. Joseph Orphan Asylum | 3 |
| 15 | 52 | Suicide | 3 |
| 16 | 14 | | |
| 17 | 23 | Total, | 415 |

### Rain Fall with Comparative Mortality, 1869 and 1870.

| | Mortality. | | | | Rain. | |
|---|---|---|---|---|---|---|
| Month | 1869 | 1870 | Increase | Decrease | 1869 | 1870 |
| January | 383 | 483 | 100 | | 1,968 | 2,665 |
| February | 333 | 421 | 88 | | 2,235 | 860 |
| March | 354 | 538 | 184 | | 1,330 | 1,880 |
| April | 381 | 495 | 114 | | 4,295 | 1,150 |
| May | 372 | 436 | 64 | | 5,670 | 800 |
| June | 434 | 720 | 286 | | 5,033 | 1,700 |
| July | 815 | 1,120 | 305 | | 3,214 | 3,710 |
| August | 1,072 | 1,037 | | 35 | 1,381 | 2,170 |
| September | 814 | 691 | | 123 | 0,890 | 2,820 |
| October | 601 | 559 | | 42 | 1,100 | 2,430 |
| November | 491 | 427 | | 64 | 2,061 | 1,160 |
| December | 440 | 415 | | 25 | 1,995 | 2,280 |
| Total | 6,490 | 7,342 | 1,141 | 289 | 31,172 | 23,625 |

1870 in excess of 1869, 852 deaths.

Money Receipts from December 25 to January 25.—Dr. Geo. H. Fuller, Delhi, Iowa, $3.00; Dr. Geo. W. Jones, Danville, Ill., 4.00; Dr. T. H. Bras, New Boston, Ill., 6.00; Dr. C. T. Hunter, Springerton, Ill., 3.00; Dr. Mary H. Thompson, City, 3.00; Dr. L. H. Humphreys, South Bend, Ind., 3.00; Dr. H. W. Kendall, Quincy, Ill., 3.00; Dr. J. F. McCormick, Fowler, Ill., 9.00; Dr. A. G. McCormick, Fowler, Ill., 3.00; Dr. Mergler, Wheeling, Ill., 6.00; Dr. G. W. Dodge, Winneconee, Wis., 3.00; Dr. T. G. Williams, Watertown, Wis., 3.00; Dr. H. C. Lester, Lincoln, Ind., 3.00; Dr. H. N. Hurlbut, City, 3.00; Dr. E. R. Willard, Wilmington, Ill., 3.00; Dr. A. C. Carr, Chesterfield, Ill., 3.00; Mrs. A. F. Hammond, City, 3.00; Dr. J. A. McDonald, Wis., 3.00; Dr. W. A. Gordon, Hannibal, Mo., 6.00; Dr. V. C. Price, Waukegan, Ill., 3.00; Dr. J. Bell, Benton Harbor, Mich., 3.00; Dr. J. Milleron, Grayville, Ill., 3.00; Dr. J. S. Bullock, Vermillion, Ill., 6.00; Drs. M. M. Sparklee & Lotta, 6.00; Dr. Wm. B Hart, Greenwood, Ill., 3.00; Dr. French, Lincoln, Neb., 6.00; Dr. Prince, Jacksonville, Ill., 3.00; Dr. O. J. Price, City, 3.00; Dr. Thos. D. Washburn, Hillsborough, Ill., 3.00; Dr. J. F. Williams, City, 3.00; Dr. J. Tenbrook, Paris, Ill., 3.00; Dr. H. C. Miner, Roanoke, Ind., 5.00; Dr. B. Lee, Marshall, Ill., 6.00; Dr. A. M. Maxwell, Friend Grove, Ill., 3.00; Dr. W. W. Grant, Davenport, Iowa, 3 00; Dr. G. W. Holder, Washburn, Wis., 3.00; Dr. T. Stephens, Winona, Minn., 12.00; Dr. A. J. Smith, City, 3.00; Dr. T. O. Bannester, Odell, Ill., 4.00; Dr. Wm. M. Landon, Burton, Ill., 6.00; Dr. A. Hagar, Marengo, Ill., 3.00; Dr. J. W. Fink, Hillsborough, Ill., 3.00; Dr. J. R. Garrell, Newton, Iowa, 5.00; Dr. J. M. Barlow, Paris, Ill., 10.50; Dr. J. P. Johnston, Lynnville, Ill., 3.00; Dr. Geo. A. Bardwell, Dixon, Ill., 3.00.

